# Health Care Costs of Firearm Injury Hospital Visits in the US

**DOI:** 10.1001/jamahealthforum.2025.3299

**Published:** 2025-09-26

**Authors:** Regina Royan, Alexander Lundberg, Ying Shan, Arielle C. Thomas, Anne M. Stey

**Affiliations:** 1 Department of Emergency Medicine, University of Michigan, Ann Arbor; 2 Buehler Center for Health Policy and Economics, Northwestern University, Chicago, Illinois; 3 Department of Surgery, Feinberg School of Medicine, Northwestern University, Chicago, Illinois; 4 Department of Surgery, Medical College of Wisconsin, Milwaukee

## Abstract

**Importance:**

Firearm injury is a leading cause of mortality in the US. Contemporary firearm injury health care costs and characteristics of hospital visits can inform investment decisions on treatment and prevention strategies.

**Objective:**

To estimate the total health care cost of new firearm injury hospital visits from 2016 to 2021 in the US.

**Design, Setting, and Participants:**

This economic evaluation study via Monte Carlo simulation included data from the Arkansas, Florida, Maryland, Massachusetts, New York, and Wisconsin Healthcare Cost and Utilization Project State Inpatient and Emergency Department databases from 2016 to 2021. Children and adults with an inpatient or emergency department (ED) hospital visit for new firearm injuries were included. Data were analyzed from June 2023 to May 2025.

**Exposures:**

Firearm-related inpatient or ED visits with new firearm injury *International Statistical Classification of Diseases and Related Health Problems, Tenth Revision* (*ICD*-*10*) diagnosis codes.

**Main Outcomes and Measures:**

A Monte Carlo simulation used new inpatient and ED firearm injury visits in 6 sample states to estimate the national health care cost for the treatment of initial firearm injuries from 2016 to 2021. The simulation also used national inpatient data from the RAND Corporation for nonsample states. Costs were adjusted for inflation to 2024 US dollars. Costs to each body region were derived from the Injury Mortality Diagnosis Matrix classification scheme.

**Results:**

The Monte Carlo analysis included 2400 simulations. Firearm injuries in the US led to an estimated 298 721 ED visits and 185 846 inpatient visits, with a total health care cost of $7.7 billion from 2016 to 2021. Inpatient admissions accounted for 93% of the cost, or $7.2 billion. Treatment for children younger than 18 years accounted for 9% of the cost, or $684 million. Annual ED and inpatient visits were both approximately stable from 2016 to 2019, at which point they grew by 42% and 40%, respectively, from 2019 to 2021. Annual total health care cost was also stable at approximately $1.2 billion until 2019, when cost began to grow to a peak of $1.6 billion in 2021. The mean (SE) ED visit cost was $1743 (4.5), and the mean (SE) inpatient admission cost was $38 879 (138.9). These costs remained stable annually over the sample period.

**Conclusions and Relevance:**

In this economic evaluation study, an increase in firearm injuries in the last 6 years paralleled an increase in costs from 2016 to 2021.

## Key Points

**Question:**

What is the total health care cost for initial firearm-related emergency department (ED) visits and inpatient hospitalizations in the US?

**Findings:**

In this economic evaluation study including data from 6 states, using a Monte Carlo simulation, firearm injuries led to an estimated 298 721 ED visits and 185 846 hospital admissions from 2016 through 2021. The total estimated firearm injury health care cost was $7.7 billion over the 6 years (in base year 2024 dollars); inpatient admissions accounted for 93% of the cost.

**Meaning:**

Child and adult firearm injury is a substantial source of health care cost in the US and has risen over time.

## Teaser

This economic evaluation study estimates the total health care cost of new firearm injury hospital visits from 2016 to 2021 in the US.

## Introduction

Firearm injury is a leading cause of disability and premature death in the US.^1^ The US Surgeon General recently described firearm injury as a public health crisis.^2^ Although costs are largest for patients with firearm injuries, the health care system also incurs financial costs in the treatment of firearm injuries.^3^ In 1990, the cost of direct expenditures for firearm injury health care and related services was $1.6 billion.^4^ Another study over 2006 to 2014 estimated combined emergency department (ED) and inpatient charges at nearly $2.8 billion annually.^5^

Contemporary cost estimates of ED and inpatient hospital firearm injury treatment can inform investment decisions on prevention strategies, such as safe storage for suicide prevention, hospital-based violence intervention programs, and trauma recovery centers, which traditionally support the population affected by intimate partner violence.^6,7,8,9,10^ Additionally, public insurance has been the most frequent source of hospital reimbursement for firearm injury health care cost. Many hospitals are reimbursed below the cost of health care provision.^11^ Hospital financial strain can threaten hospitals’ ability to provide care for all medical conditions.^12,13,14^

This study estimated mean and total health care costs of ED and inpatient visits for new firearm injuries in the US from 2016 to 2021. These costs were stratified by year, visit type, patient age, injury body location, payer, hospital bed size, and trauma center level designation, among other factors, to understand where hospitals account for the greatest health care cost.

## Methods

### Study Design

This economic analysis used observational administrative data from 2016 to 2021 to estimate total firearm injury ED and inpatient visit costs in the US. The study followed both the Strengthening the Reporting of Observational Studies in Epidemiology (STROBE) reporting guideline and Consolidated Health Economic Evaluation Reporting Standards (CHEERS) reporting guideline for economic evaluations.^15,16^ This research was deemed exempt by the Northwestern University Institutional Review Board because it was not human subjects research.

### Data Sources

Primary data sources were the Arkansas, Florida, Maryland, Massachusetts, New York, and Wisconsin Statewide Inpatient Databases (SID) and State Emergency Department Databases (SEDD) maintained by the Agency for Healthcare Research and Quality Healthcare Cost and Utilization Project.^17^ Data from SEDD/SID for the 6 sample states were linked via hospital identifiers to American Hospital Association (AHA) Annual Survey data.^17,18^ The RAND Corporation state-level database provided estimates of inpatient firearm injury hospitalization rates for nonsample states via personal communication.

### Inclusion Criteria

The sample included any ED and inpatient visits in SEDD/SID with a new firearm injury identified by an *International Statistical Classification of Diseases and Related Health Problems, Tenth Revision* (*ICD*-*10*) code in eTable 1 in Supplement 1 with a final character *A* to indicate a new visit in Arkansas, Florida, Maryland, Massachusetts, New York, and Wisconsin from 2016 to 2021. Patients of all ages and injuries of any intent were included. Cost estimates were derived from 74 619 cases with nonmissing charge values. Visits with a missing charge value were excluded (n = 15 193). Costs across variable categories may not sum to total cost due to missing values.

### Cost Perspective

This study adopted a hospital cost perspective. Hospitals bear a large financial cost of initial firearm injury because many patients with firearm injuries are uninsured or underinsured. Furthermore, hospitals face increased calls to address social drivers of health.^19^

### Main Outcome Measurement

The primary outcome was total health care visit cost, obtained through the product of the total visit charge and the hospital-specific, year-specific cost to charge ratio. Costs were converted to 2024 US dollar values with Consumer Price Index data from the Federal Reserve Bank of Minneapolis.^20^

Patient demographic characteristics included age, sex, race and ethnicity, payer type, home zip code income quartile, and discharge disposition. Age was grouped into categories of age 0 to 17 years, age 18 to 24 years, age 25 to 44 years, age 45 to 64 years, and 65 years or older. Sex was categorized as male or female. Race and ethnicity categories in this database were abstracted from the medical record from Healthcare Cost and Utilization Project categories and included Asian or Pacific Islander, Black, Hispanic, Native American, White, and other race. Payer included Medicaid, Medicare, private, self-pay, no charge, and other (workers’ compensation, TRICARE, Civilian Health and Medical Program of the Department of Veterans Affairs, Title V, and other government programs). Home zip code income quartiles were defined by yearly national median income thresholds in SID/SEDD. Discharge disposition included routine home, transfer to acute care hospital, transfer to other facility (hospice, rehabilitation, long-term care hospital, psychiatric hospital, skilled nursing facility, or intermediate care facility), home health care, against medical advice, and died.

Injury variables included intent, body region, severity, comorbidities, and reinjury. Intent was obtained from *ICD*-*10* codes and included assault, self-inflicted, unintentional injury, undetermined, and legal intervention. Injury body region was derived from *ICD*-*10* codes with the Injury Mortality Diagnosis Matrix.^21^ Injury severity score (ISS) was defined as the squared and summed Abbreviated Injury Scale score using the ICD Programs for Injury Categorization.^22^ ISS was categorized as mild (score of 0 to 8), serious (score of 9 to 25), and severe (score of 26 to 75). Patient comorbidities were derived with the Weighted Elixhauser Index of Comorbidity, then categorized as none, 1, 2, and 3 or more.^23,24^ Reinjury was defined as patients who presented for 2 new firearm injuries to an acute care hospital in the same calendar year.^25^ Reinjury was classified as either no reinjury or at least 1 reinjury.

Hospital AHA variables included number of inpatient beds, the ratio of Medicaid discharges to all-payer discharges, core-based statistical area (CBSA) designation, trauma center level designation, rural hospital designation, total inpatient admissions, total ED visits, and medical school affiliation. The inpatient hospital beds category was grouped into 1 to 99, 100 to 499, and 500 or more beds. Hospitals’ percentage of Medicaid discharges were divided into ascending quartiles from total hospital discharges for all conditions (calculated from the sample in each simulation). Hospitals’ CBSA was categorized as metropolitan, micropolitan, and rural. Hospitals’ trauma center level designation was categorized as regional resource (level I), community (level II), rural (level III), and greater (level IV). Hospitals’ total inpatient admissions were categorized into ascending quartiles based on all hospital admissions. Hospitals’ total ED visits were categorized into ascending quartiles based on all ED visits. Rural hospital status and medical school affiliation were binary variables in the AHA dataset.

### Statistical Analysis

A Monte Carlo simulation estimated the national cost of ED visits and inpatient admissions for new firearm injuries based on patient claims data from Arkansas, Florida, Maryland, Massachusetts, New York, and Wisconsin. The simulation used RAND Corporation yearly state-level firearm injury inpatient admissions estimates for other states.^26^ The Monte Carlo procedure (1) drew a pure random sample, with replacement, of inpatient admissions from the 6 sample states in each year equal to the size of inpatient admissions in nonsample states; (2) drew an analogous sample for ED visits, with the number of ED visits determined by the product of RAND inpatient admission estimates and the ratio of ED visits to inpatient hospitalizations in the 6 sample states; and (3) combined the resampled data with original data to create a national, annual sample from 2016 to 2021.

While previous national studies used stratified and weighted samples of approximately 20% of hospitals nationwide,^27,28,29^ the National Inpatient Sample,^11,30,31,32^ or Nationwide Emergency Department Sample^5^ to estimate firearm injury health care costs, the Monte Carlo simulation offers 2 benefits.^33^ First, the state data contained a VisitLink identifier to track individuals across visits. Second, the AHA linkage contained additional hospital variables, such as trauma center designation and admissions by payer type, which are unavailable in the national sample. Cost estimates were derived as mean values across the simulations. All analysis was conducted in SAS Studio version 9.4 (SAS Institute) and R version 4.4.1 (The R Foundation). Simulation R code is available in the eMethods in Supplement 1. Data were analyzed from June 2023 to May 2025.

## Results

From 2016 to 2021, an estimated 484 567 initial firearm injury hospital visits occurred nationally (Table 1).^34^ Of them, 298 721 were ED visits, and 185 846 involved an inpatient hospital admission. Using a Monte Carlo analysis including 2400 simulations, these visits incurred an estimated $7.7 billion total cost, 93% of which was attributable to inpatient admissions. The mean (SE) cost per inpatient visit was $38 879 (138.9), and the mean (SE) cost per ED visit was $1743 (4.5) (Table 2).

**Table 1.** Monte Carlo Estimates for Total Cost of Emergency Department and Inpatient Visits for New Firearm Injuries in the US from 2016 to 2021

Characteristic	Total cost, $ (SE)^a^		
	Overall (N = 484 567)	Emergency department (n = 298 721)	Inpatient (n = 185 846)
Overall	7746.3 (25.9)	520.7 (1.3)	7225.0 (25.8)
Age, y			
0-17	684.4 (10.1)	44.7 (0.5)	639.8 (10.1)
18-24	2083.1 (14.3)	154.2 (0.8)	1928.9 (14.3)
25-44	3668.6 (19.6)	237.1 (1.0)	3431.5 (19.6)
45-64	1014.5 (10.3)	62.8 (0.6)	951.6 (10.3)
≥65	286.7 (5.4)	18.5 (0.3)	268.2 (5.4)
Sex			
Female	855.6 (9.2)	66.4 (0.6)	789.2 (9.1)
Male	6889.7 (25.2)	454.0 (1.3)	6435.7 (25.2)
Race and ethnicity^b^			
Asian or Pacific Islander	37.1 (1.8)	2.7 (0.1)	34.4 (1.8)
Black	4513.7 (21.2)	321.7 (1.2)	4191.9 (21.2)
Hispanic	888.2 (10.8)	54.8 (0.5)	833.4 (10.8)
Native American	11.8 (2.0)	0.9 (0.1)	11.0 (2.0)
White	1737.2 (13.2)	108.9 (0.6)	1628.3 (13.2)
Other race	317.6 (7.2)	14.4 (0.3)	303.2 (7.1)
Payer			
Medicaid	4004.3 (23.2)	203.2 (1.0)	3801.1 (23.2)
Medicare	446.0 (6.5)	23.3 (0.3)	422.6 (6.4)
Private	1514.5 (12.7)	93.8 (0.7)	1420.7 (12.7)
Self-pay	1057.5 (7.6)	163.1 (0.8)	894.4 (7.6)
No charge^c^	196.0 (3.3)	7.0 (0.1)	189.0 (3.3)
Other^d^	512.4 (6.9)	28.4 (0.4)	484.0 (6.9)
Home zip code income quartile^e^			
1	4021.0 (20.5)	267.7 (1.0)	3753.3 (20.4)
2	1718.7 (13.1)	111.3 (0.7)	1607.4 (13.1)
3	1147.8 (12.0)	80.9 (0.6)	1066.9 (12.0)
4	609.3 (9.4)	39.7 (0.5)	596.6 (9.4)
Discharge disposition			
Routine home	3919.3 (14.7)	356.1 (1.0)	3563.2 (14.7)
Transfer to acute care hospital	286.8 (6.7)	41.9 (0.3)	244.9 (6.7)
Transfer to other facility^f^	1759.7 (19.2)	16.4 (0.3)	1743.3 (19.2)
Home health care	998.1 (11.6)	2.6 (0.1)	995.6 (11.6)
Against medical advice	128.9 (2.8)	10.2 (0.2)	118.7 (2.8)
Died	642.3 (8.6)	91.0 (1.0)	551.4 (8.6)
Injury intent			
Assault	3695.2 (21.2)	203.3 (1.0)	3149.9 (21.1)
Self-inflicted	618.9 (8.4)	16.5 (0.3)	602.5 (8.4)
Unintentional	3145.4 (16.9)	276.3 (1.0)	2869.0 (16.8)
Undetermined	178.3 (3.8)	20.2 (0.3)	158.1 (3.8)
Legal intervention	108.5 (3.5)	4.5 (0.2)	104 (3.5)
Body region			
Head and neck	327.0 (5.6)	38.8 (0.4)	288.2 (5.6)
Face	112.1 (3.1)	7.7 (0.2)	104.4 (3.1)
Chest	285.7 (5.0)	37.2 (0.5)	248.5 (5.0)
Abdomen	785.8 (9.8)	46.3 (0.5)	739.5 (9.8)
Extremities	1426.3 (7.1)	227.2 (0.8)	1199.1 (7.1)
Multiple regions	4724.4 (25.2)	155.2 (1.0)	4569.2 (25.2)
Other^g^	11.7 (0.6)	2.3 (0.1)	9.5 (0.6)
Injury severity score			
0-8	1414.9 (6.6)	315.7 (0.9)	1099.2 (6.6)
9-25	3618.2 (17.6)	164.9 (1.0)	3453.3 (1735.0)
26-75	2713.1 (22.2)	40.0 (0.6)	2673.1 (22.2)
Elixhauser Comorbidity Score			
0	1852.1 (7.6)	379.3 (1.1)	1472.8 (7.5)
1	1646.8 (10.1)	96.9 (0.8)	1549.9 (10.1)
2	1476.8 (12.4)	31.3 (0.5)	1445.4 (12.4)
≥3	2770.6 (23.5)	13.1 (0.3)	2757.5 (23.5)
Reinjury^h^			
0	7052.9 (25.5)	463.1 (1.3)	6589.8 (25.5)
≥1	693.4 (7.5)	57.5 (0.5)	635.8 (7.5)
Hospital beds			
1-99	47.7 (1.1)	23.2 (0.2)	24.5 (1.1)
100-499	2491.0 (15.1)	207.6 (0.9)	2283.4 (15.0)
≥500	5208.1 (23.5)	289.5 (1.1)	4918.6 (23.5)
Percentage of hospital Medicaid discharges quartile^i^^,^^j^			
1	1422.6 (30.0)	107.7 (1.3)	1314.9 (29.0)
2	1638.5 (29.9)	130.3 (1.3)	1508.2 (28.9)
3	2523.3 (44.0)	135.3 (0.8)	2388.0 (44.1)
4	2162.4 (43.1)	146.9 (0.8)	2015.4 (43.1)
Core-based statistical area			
Metro	7690.5 (25.6)	500.2 (1.3)	7190.3 (25.5)
Micro	44.8 (1.3)	11.9 (0.2)	32.9 (1.3)
Rural	11.5 (0.3)	8.2 (0.1)	3.3 (0.3)
Level of trauma center			
Regional resource (level I)	4737.5 (23.5)	260.3 (1.1)	4477.2 (23.4)
Community (level II)	1818.8 (13.1)	108.8 (0.7)	1709.9 (13.1)
Rural (level III)	178.4 (2.7)	32.2 (0.3)	146.2 (2.7)
Greater (level IV)	12.5 (0.4)	8.6 (0.1)	3.8 (0.3)
Rural hospital			
No	6597.7 (25.3)	441.9 (1.3)	6155.8 (25.2)
Yes	405.6 (6.3)	27.5 (0.3)	378.1 (6.3)
Total inpatient admissions quartile^j^			
1	861.8 (32.3)	127.1 (1.7)	734.7 (30.8)
2	1817.3 (29.9)	103.7 (1.4)	1713.7 (28.8)
3	2346.6 (47.7)	156.4 (2.1)	2190.3 (54.8)
4	2721.0 (51.4)	133.1 (2.3)	2587.9 (49.4)
Total emergency department visits quartile^j^			
1	1203.2 (34.2)	105.3 (1.0)	1097.9 (33.3)
2	1906.9 (34.4)	143.9 (1.1)	1763.0 (33.8)
3	2097.5 (32.1)	138.3 (1.5)	1959.2 (31.2)
4	2539.1 (33.2)	132.7 (1.3)	2406.4 (32.3)
Medical school affiliation			
No	1455.5 (10.2)	120.4 (0.6)	1335.2 (10.2)
Yes	6291.2 (25.1)	399.9 (1.3)	5891.3 (25)

^a^ Dollars are inflation adjusted to base year 2024.

^b^ Race and ethnicity categories in this database were abstracted from the medical record from Healthcare Cost and Utilization Project categories and included Asian or Pacific Islander, Black, Hispanic, Native American, White, and other race.

^c^ Arkansas and Wisconsin classify no charge as self-pay.

^d^ Other insurance includes worker’s compensation, TRICARE, Civilian Health and Medical Program of the Department of Veterans Affairs, Title V, and other government programs.

^e^ Zip code income quartile is a quartile classification of the estimated median household income of residents in the patient’s zip code and was defined yearly.^34^

^f^ Other type of facility includes discharge to hospice, rehabilitation, long-term care hospital, psychiatric hospital, skilled nursing facility, or intermediate care facility.

^g^ Other includes those unclassifiable by body site using the Barell Matrix.

^h^ Reinjury is defined as the number of times a patient presented during the year for a new firearm injury.

^i^ Hospital Medicaid percentage is calculated as the ratio of Medicaid discharges to total discharges in ascending order.

^j^ Threshold values for the quartiles were calculated in each simulation.

**Table 2.** Monte Carlo Estimates for Mean Hospital Cost per Emergency Department and Inpatient Visits for New Firearm Injuries in the US from 2016 to 2021

Characteristic	Cost, mean (SE), $^a^	
	Emergency department (n = 298 721)	Inpatient (n = 185 846)
Overall	1743.0 (4.5)	38 879.0 (138.9)
Age, y		
0-17	1623.0 (14.7)	42 116.0 (574.1)
18-24	1739.0 (7.3)	36 996.0 (240.4)
25-44	1791.0 (7.0)	40 185.0 (209.7)
45-64	1700.0 (12.3)	38 449.0 (355.6)
≥65	1552.0 (18.1)	32 741.0 (544.3)
Sex		
Female	1651.0 (12.3)	35 052.0 (341.3)
Male	1757.0 (4.8)	39 408.0 (149.9)
Race and ethnicity^b^		
Asian or Pacific Island	1650.0 (48.7)	39 343.0 (1579.8)
Black	1853.0 (6.2)	39 961.0 (185.3)
Hispanic	1741.0 (13.2)	39 960.0 (438.1)
Native American	1825.0 (117.7)	37 634.0 (6452.0)
White	1418.0 (6.9)	35 240.0 (244.6)
Other race	1909.0 (25.2)	44 049.0 (910.9)
Payer		
Medicaid	1889.0 (7.6)	46 515.0 (252.5)
Medicare	1470.0 (14.1)	34 031.0 (423.6)
Private	1613.0 (10.2)	37 442.0 (285.8)
Self-pay	1713.0 (7.4)	26 949.0 (182.8)
No charge^c^	1370.0 (21.2)	26 771.0 (343.4)
Other^d^	1804.0 (23.2)	37 214.0 (425.4)
Home zip code income quartile^e^		
1	1734.0 (5.6)	38 915.0 (191.4)
2	1654.0 (8.4)	36 756.0 (263.3)
3	1825.0 (10.9)	39 423.0 (377.0)
4	1742.0 (19.4)	43 935.0 (614.9)
Discharge disposition		
Routine home	1636.0 (4.3)	28 674.0 (109.6)
Transfer to acute care hospital	1398.0 (8.5)	58 058.0 (1334.1)
Transfer to other facility^f^	1649.0 (20.4)	87 113.0 (757.6)
Home health care	3646.0 (155.0)	56 209.0 (519.0)
Against medical advice	1609.0 (24.5)	24 078.0 (465.0)
Died	2752.0 (25.0)	37 898.0 (508.1)
Injury intent		
Assault	2192.0 (8.8)	43 392.0 (236.7)
Self-inflicted	2067.0 (25.4)	40 159.0 (458.8)
Unintentional	1504.0 (5.2)	34 444.0 (183.4)
Undetermined	1828.0 (17.7)	31 112.0 (617.4)
Legal intervention	1390.0 (41.5)	52 196.0 (1311.6)
Body region		
Head and neck	1659.0 (11.5)	32 062.0 (519.6)
Face	1511.0 (30.5)	35 935.0 (825.2)
Chest	2065.0 (22.8)	30 871.0 (516.4)
Abdomen	2345.0 (23.4)	38 911.0 (439.5)
Extremities	1375.0 (4.5)	20 944.0 (99.6)
Multiple regions	2668.0 (13.3)	53 856.0 (259.2)
Other^g^	1049.0 (39.0)	11 746.0 (632.8)
Injury severity score		
0-8	1508.0 (4.2)	18 513.0 (91.3)
9-25	2156.0 (10.6)	38 270.0 (170.9)
26-75	3114.0 (38.4)	73 772.0 (496.3)
Elixhauser Comorbidity Score		
0	1616.0 (4.5)	21 919.0 (92.1)
1	2104.0 (13.9)	31 382.0 (165.1)
2	2395.0 (31.0)	44 730.0 (310.1)
≥3	2694.0 (47.2)	74 626.0 (521.0)
Reinjury^h^		
0	1764.0 (4.8)	39 586.0 (147.9)
≥1	1594.0 (10.9)	32 808.0 (325.9)
Hospital beds		
1-99	1010.0 (7.3)	20 093.0 (653.5)
100-499	1393.0 (5.1)	31 562.0 (183.9)
≥500	2294.0 (7.8)	43 815.0 (193.0)
Percentage of hospital Medicaid discharges quartile^i^^,^^j^		
1	1342.0 (7.9)	32 271.0 (243.5)
2	1703.0 (9.8)	33 894.0 (234.0)
3	2061.0 (11.3)	43 163.0 (305.5)
4	1940.0 (8.4)	44 540.0 (358.4)
Core-based statistical area		
Metro	1797.0 (4.5)	39 080.0 (138.8)
Micro	963.0 (9.3)	20 184.0 (637.9)
Rural	1084.0 (12.4)	15 919.0 (932.7)
Level of trauma center		
Regional resource (level I)	2484.0 (9.1)	45 658.0 (215.6)
Community (level II)	1840.0 (9.5)	35 053.0 (229.6)
Rural (level III)	1136.0 (8.5)	20 802.0 (291.0)
Greater (level IV)	1040.0 (11.2)	14 416.0 (1002.5)
Rural hospital		
No	1824.0 (5.0)	40 077.0 (157.8)
Yes	1493.0 (11.9)	35 518.0 (489.9)
Total inpatient admissions quartile^j^		
1	1292.0 (7.1)	32 440.0 (383.3)
2	1494.0 (10.5)	33 200.0 (233.4)
3	2338.0 (21.9)	40 474.0 (333.4)
4	2095.0 (15.2)	45 047.0 (295.2)
Total emergency department visits quartile^j^		
1	1262.0 (6.4)	29 205.0 (238.0)
2	1907.0 (9.7)	38 726.0 (299.3)
3	1885.0 (14.2)	41 145.0 (331.9)
4	2014.0 (14.3)	43 688.0 (350.8)
Medical school affiliation		
No	1138.0 (4.4)	27 422.0 (149.1)
Yes	2079.0 (6.0)	42 960.0 (173.5)

^a^ Dollars are inflation adjusted to base year 2024.

^b^ Race and ethnicity categories in this database were abstracted from the medical record from Healthcare Cost and Utilization Project categories and included Asian or Pacific Islander, Black, Hispanic, Native American, White, and other race.

^c^ Arkansas and Wisconsin classify no charge as self-pay.

^d^ Other insurance includes worker’s compensation, TRICARE, Civilian Health and Medical Program of the Department of Veterans Affairs, Title V, and other government programs.

^e^ Zip code income quartile is a quartile classification of the estimated median household income of residents in the patient’s zip code and was defined yearly.^34^

^f^ Other type of facility includes discharge to hospice, rehabilitation, long-term care hospital, psychiatric hospital, skilled nursing facility, or intermediate care facility.

^g^ Other includes those unclassifiable by body site using the Barell Matrix.

^h^ Reinjury is defined as the number of times a patient presented during the year for a new firearm injury.

^i^ Hospital Medicaid percentage is calculated as the ratio of Medicaid discharges to total discharges in ascending order.

^j^ Threshold values for the quartiles were calculated in each simulation.

### Temporal Trends

**Figure 1.**
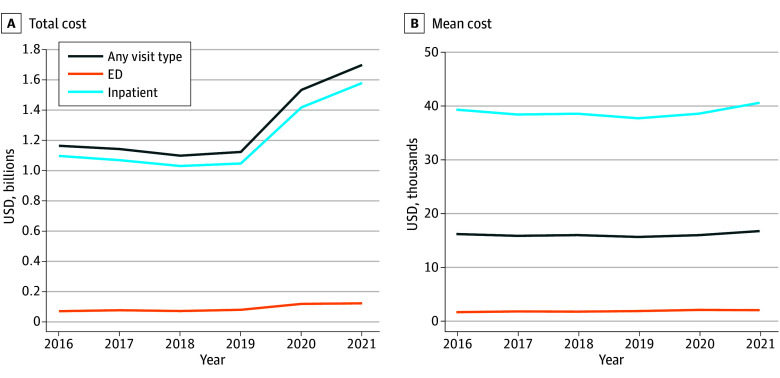
National Monte Carlo Estimates of Total Cost and Mean Cost per Visit for Firearm Injury Emergency Department (ED) Visits and Inpatient Hospitalizations by Visit Type from 2016 to 2021

Figure 1A shows annual trends in total costs for inpatient and ED visits. The annual aggregate cost remained at approximately $1.2 billion until 2020, at which point cost rose to a peak of $1.6 billion in 2021. Figure 1B presents annual trends in the mean costs of inpatient and ED visits, which were approximately constant across the sample period.

**Figure 2.**
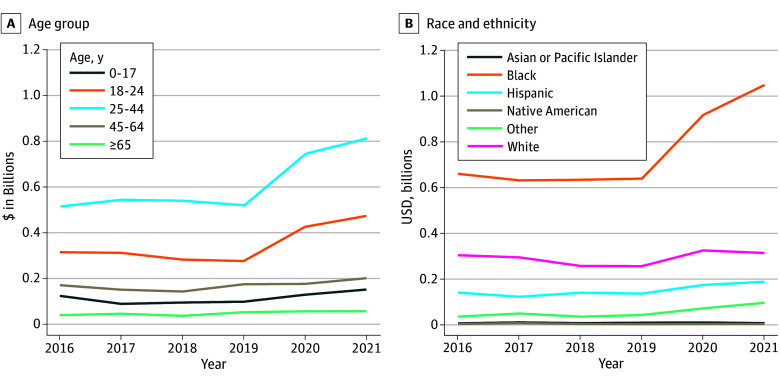
National Monte Carlo Estimates of Total Cost for Firearm Injury Emergency Department Visits and Inpatient Hospitalizations by Age Group and Race and Ethnicity from 2016 to 2021

Figure 2A shows annual trends in total health care costs by age group. For children aged 0 to 17 years, costs declined by 28% from 2016 to 2017, remained roughly constant from 2017 to 2019, then increased by 54% from 2019 to 2021. Costs for patients aged 18 to 24 years and aged 25 to 44 years remained stable until 2019, rising 72% and 56%, respectively, by 2021. Total costs for patients aged 45 to 64 years or 65 years or older remained largely flat over the sample period. Figure 2B presents cost trends by race and ethnicity, which were approximately flat for each group until 2019. From 2019 to 2021, Asian or Pacific Islander patients experienced an increase of 73%; Black patients, an increase of 64%; Hispanic patients, an increase of 39%; and White patients, an increase of 23%.

### Patient Variables

#### Total Cost

Nationally, firearm injury health care costs were most heavily concentrated among male children and adults who were Black, had Medicaid as payer, and had residence in lower-income zip codes. From 2016 to 2021, initial firearm injury treatment incurred an estimated $6.9 billion in cost for male patients. Total hospital costs for caring for Black patients were $4.5 billion. Hospital costs for White patients were the next highest at $1.7 billion, followed by Hispanic patients at $0.9 billion, non-Hispanic patients of another race at $0.3 billion, and Asian or Pacific Islander patients and Native American patients at $0.04 billion and $0.01 billion, respectively. Hospital costs of care were $4.0 billion for patients with Medicaid as payer, $1.5 billion for those with private insurance, $1.1 billion for those who self-paid, $0.5 billion for those with other insurance, $0.4 billion for those with Medicare, and $0.2 billion for those who were not charged. Hospitals costs of care for patients from a home zip code in the lowest income quartile was $4.0 billion, followed by $1.7 billion for those from a home in the second quartile, $1.1 billion for those from a home in the third quartile, and $0.6 billion for those from a home in the fourth quartile.

Injuries categorized as assaults or unintentional accounted for the largest proportion of firearm injury health care cost. Assaults accounted for $3.7 billion, while injuries categorized as unintentional accounted for $3.1 billion. Injuries to multiple body regions totaled $4.7 billion and accounted for 31.6% of ED costs and 64.4% of inpatient costs (eFigure in Supplement 1). Injuries to isolated extremities totaled $1.4 billion and accounted for 40.4% of ED costs and 15.4% of inpatient costs. Lastly, serious injuries with an ISS from 9 to 25 accounted for $3.6 billion, followed by severe injuries with an ISS from 26 to 75 at $2.7 billion.

#### Mean Cost

Mean (SE) inpatient admission cost for assaults was $43 392 (236.7), followed by $40 159 (458.8) for self-inflicted injuries and $34 444 (183.4) for injuries categorized as unintentional. Mean (SE) inpatient cost was highest for firearm injury to multiple body regions at $53 856 (259.2). The second highest body region cost category was abdominal injuries at $38 911 (439.5). Mean (SE) inpatient cost was highest for severe firearm injuries with an ISS of 26 to 75 at $73 772 (496.3) compared with serious firearm injuries with an ISS of 9 to 25 at $38 270 (170.9) and minor injuries with an ISS of 0 to 8 at $18 513 (91.3). Because the cost distribution can be skewed by a small number of relatively expensive visits, median values may provide a better sense of cost for typical visits than mean values (eTable 2 in Supplement 1).

### Hospital Variables

#### Total Cost

Over the sample period, hospitals with 500 or more beds saw a total cost of $5.2 billion, and hospitals with 100 to 499 beds saw a cost of $2.5 billion. Costs ranged from a high of $2.1 billion for hospitals in the upper quartile of percentage of Medicaid discharges to a low of $1.4 billion for those in the lowest quartile. Almost all costs were concentrated in hospitals in a metro CBSA. Regional resource hospitals (level I trauma center) accounted for the highest total of new firearm injury costs at $4.7 billion, followed by community hospitals (level II trauma center) at $1.8 billion. Lastly, hospitals affiliated with a medical school accounted for $6.3 billion new firearm injury cost.

#### Mean Cost

Mean (SE) cost ranged from a high of $43 815 (193.0) for hospitals with 500 or more beds to a low of $20 093 (653.5) for hospitals with 1 to 99 beds. Mean (SE) cost ranged from a high of $44 540 (358.4) for hospitals in the upper quartile of percentage of Medicaid discharges to a low of $32 271 (243.5) for hospitals in the first quartile. The mean (SE) cost ranged from a high of $45 648 (215.6) for regional resource hospitals (level I) to a low of $14 416 (1002.5) for greater nontrauma hospitals (level IV). See eTable 2 in Supplement 1 for median costs.

### Mean Inpatient Cost and Percentage of Contribution to Total Cost

**Figure 3.**
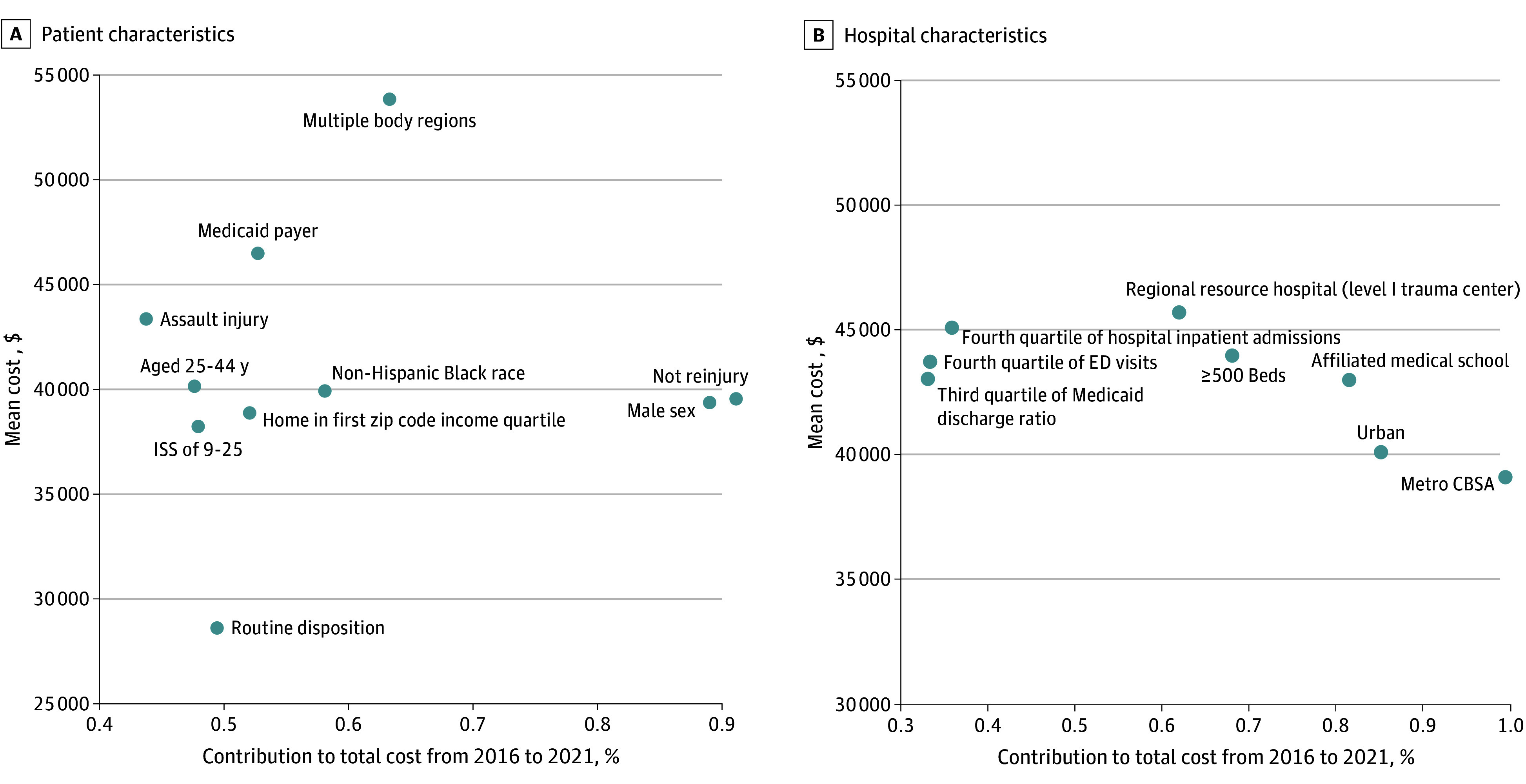
Mean Cost per Inpatient Admission of New Firearm Injury for Patient and Hospital Characteristics With Top Percentage Contributions to National Inpatient Cost from 2016 to 2021 CBSA indicates core-based statistical area; ED, emergency department; ISS, injury severity score.

Figure 3 summarizes the highest contributions to overall cost via a scatter plot with the mean inpatient treatment cost for the highest 10 categories of percentage contributions to total cost. Figure 3A displays patient characteristics and Figure 3B displays hospital characteristics. For example, injuries to multiple body regions accounted for 63% of total cost, and admissions for these injuries incurred a mean (SE) cost of $53 856 (259.2).

## Discussion

US hospital health care costs for new firearm injuries were an estimated $7.7 billion from 2016 to 2021. Annual costs increased from $1.2 billion to $1.6 billion from 2019 to 2021. The increase coincided with a rise in violent crime during the COVID-19 pandemic.^35,36^ Although total costs increased, mean cost per visit remained largely constant from 2016 to 2021. Firearm injury incidence therefore appears to be a primary driver of the rise in costs.

Another contribution of this study is the estimation of costs in pediatric populations, as the incidence and mortality from pediatric firearm injuries have increased in recent years.^37,38^ From 2016 to 2021, firearm injuries incurred approximately $684 million in hospital costs for patients younger than 18 years. The annual cost of treatment for pediatric patients also grew by an estimated 54% from 2019 to 2021. Additionally, the proportion of all costs caused by injuries categorized as unintentional grew from 40% in 2016 to 52% in 2021. Future research should explore explanations for the rise.

Black male children and adults with a home zip code in the lowest income quartile with multiple assault injuries presenting to large, urban regional resource hospitals (level I trauma centers) serving the largest proportion of Medicaid patients accounted for the largest proportion of firearm health care costs. Assault injuries accounted for most costs. However, intent is often coded inaccurately in hospital systems, and many injuries coded as unintentional were likely intentional in reality.^27,39^

Firearm reinjury has been found to occur in up to 40% of firearm injury survivors.^40,41^ In this study, reinjury estimates were biased downward because the ability to track the same patient ends at each calendar year. Despite the bias, subsequent visits accounted for 9% of the national cost estimate. Reinjury may offer a prevention point for injury and cost reduction if risk factors for violence can be modified through hospital-based violence intervention programs. These programs appear to be cost-effective.^8,10,42,43^ These programs may also be a potential focal point for cost reduction because average costs of treatment did not rise over the sample period. Rather, changes in incidence appear to explain the increase in cost.

Across hospital characteristics, firearm injury health care costs were borne primarily by hospitals with 500 or more beds and in the upper quartile of percentage of Medicaid discharges. These results are consistent with a separate study by Okeke et al,^31^ who used the Nationwide Inpatient Sample and found that moderate to high safety net mixed hospitals bore the highest costs across a longer period from 2003 to 2020. Payer type is an important consideration for hospitals and policymakers. Because hospitals are on average reimbursed below cost by most Medicaid programs, high rates of Medicaid and uninsurance can put hospitals under financial strain and threaten the ability to provide care.^44,45,46,47,48^ Notably, however, Medicaid expansion can promote financial viability through the conversion of uninsurance to Medicaid.^49^

Okeke et al^31^ offer perhaps the most closely related study. The authors estimated $1.4 billion in total inpatient costs for the year 2020, which matches our own estimate in Figure 1. While our study used a Monte Carlo simulation with state data, Okeke et al^31^ used the national inpatient sample. The concordance in estimates across the 2 approaches offers encouragement for the accuracy in addition to the rigor and reproducibility of our results.

Although $7.7 billion in total costs over the sample period conveys a substantial source of US health care cost, the complete financial cost of firearm injury treatment is certainly larger. The administrative data do not include physician professional fees, which increases cost by approximately 20%.^50^ Likewise, these data omit prehospital transportation cost, such as the ambulance or helicopter costs, nor do they include trauma activation fees.^51^ Additionally, the cost of readmission or a subsequent visit for the same firearm injury can be high. These visits were not included in this study. One study estimated a national cost of $54.2 million for readmission among adults.^52^ Another study found nonfatal firearm injury survivors experienced increased health care use.^53^ Furthermore, only acute care hospital costs were included, not postacute care, such as long-term nursing care, rehabilitation, and physical therapy. Initial hospitalization costs only account for 60% of total costs in the first year of a firearm injury.^54^ Finally, indirect costs, such as caregiver expenses and lost wages, were also not included but are known to be substantial.^54^

### Limitations

This study was subject to several limitations. First, the sample states with SEDD/SID data may not perfectly represent the demographic composition of the US. Bias was mitigated through the use of geographically and demographically diverse states as well as inpatient data for nonsample states in the simulation. Second, the VisitLink identifier used to estimate reinjury was restricted by calendar year, so censored values create underestimates of reinjury. Third, acute care hospital costs are only a portion of firearm injury cost to the health care system, which also include prehospital health care professional costs, professional fees, postacute care costs, and other direct care costs. Fourth, this study adopted the perspective of health care cost and does not estimate firearm injury costs borne by survivors, their caregivers, or their communities.

## Conclusions

In this economic evaluation study, new firearm injuries created an estimated $7.7 billion in ED-only and inpatient hospital health care costs from 2016 to 2021. These data can inform decision-makers who prioritize firearm injury prevention strategies at the patient level. The data can also inform policies to support the hospitals disproportionally affected by firearm injury costs. As mean costs per visit have remained approximately constant over time, efforts to prevent firearm injury may hold potential as cost-saving measures to reduce uncompensated care for health systems and costs to government-sponsored health care plans.

## Notes

Supplement 1. **eMethods.** Monte Carlo Simulation R Code **eTable 1.** List of *International Statistical Classification of Diseases and Related Health Problems, Tenth Revision* Codes to Identify Firearm Injury Patients **eTable 2.** Monte Carlo Estimates for Median Hospital Cost per Emergency Department and Inpatient Visits for New Firearm Injuries in the US from 2016 to 2021 **eFigure.** National Monte Carlo Estimates of Mean Cost for Firearm Injury Emergency Department Visits and Inpatient Hospitalizations by Body Region

Supplement 2. Data Sharing Statement

## References

[1] Fowler KA, Dahlberg LL, Haileyesus T, Annest JL. Firearm injuries in the United States. Prev Med. 2015;79:5-14. doi:10.1016/j.ypmed.2015.06.002 26116133 PMC4700838

[2] Abbasi J, Hswen Y. US Surgeon General Vivek Murthy: firearm violence is a public health crisis. JAMA. 2024;332(6):439-441. doi:10.1001/jama.2024.2485 39028642

[3] Miller GF, Barnett SBL, Florence CS, McDavid Harrison K, Dahlberg LL, Mercy JA. Costs of fatal and nonfatal firearm injuries in the U.S., 2019 and 2020. Am J Prev Med. 2024;66(2):195-204. doi:10.1016/j.amepre.2023.09.026 38010238 PMC10843794

[4] Max W, Rice DP. Shooting in the dark: estimating the cost of firearm injuries. Health Aff (Millwood). 1993;12(4):171-185. doi:10.1377/hlthaff.12.4.171 7802749

[5] Gani F, Sakran JV, Canner JK. Emergency department visits for firearm-related injuries in the United States, 2006–14. Health Aff (Millwood). 2017;36(10):1729-1738. doi:10.1377/hlthaff.2017.0625 28971917

[6] Riley GF. Administrative and claims records as sources of health care cost data. Med Care. 2009;47(7)(suppl 1):S51-S55. doi:10.1097/MLR.0b013e31819c95aa 19536019

[7] Romo ND, Dawkins-Hamilton C, Confino M. The potential impact of hospital violence intervention programs. Hosp Pediatr. 2024;14(11):e497-e499. doi:10.1542/hpeds.2024-007816 39444372

[8] Juillard C, Cooperman L, Allen I, . A decade of hospital-based violence intervention: benefits and shortcomings. J Trauma Acute Care Surg. 2016;81(6):1156-1161. doi:10.1097/TA.0000000000001261 27653168

[9] Gottlieb LM, Wing H, Adler NE. A systematic review of interventions on patients’ social and economic needs. Am J Prev Med. 2017;53(5):719-729. doi:10.1016/j.amepre.2017.05.011 28688725

[10] Juillard C, Smith R, Anaya N, Garcia A, Kahn JG, Dicker RA. Saving lives and saving money: hospital-based violence intervention is cost-effective. J Trauma Acute Care Surg. 2015;78(2):252-257. doi:10.1097/TA.0000000000000527 25757108

[11] Peek-Asa C, Butcher B, Cavanaugh JE. Cost of hospitalization for firearm injuries by firearm type, intent, and payer in the United States. Inj Epidemiol. 2017;4(1):20. doi:10.1186/s40621-017-0120-0 28721637 PMC5515719

[12] American Hospital Association. America’s hospitals and health systems continue to face escalating operational costs and economic pressures as they care for patients and communities. Accessed February 2, 2025. https://www.aha.org/system/files/media/file/2024/05/Americas-Hospitals-and-Health-Systems-Continue-to-Face-Escalating-Operational-Costs-and-Economic-Pressures.pdf

[13] Goldstick JE, Zeoli A, Mair C, Cunningham RM. US firearm-related mortality: national, state, and population trends, 1999–2017. Health Aff (Millwood). 2019;38(10):1646-1652. doi:10.1377/hlthaff.2019.00258 31589525 PMC7028356

[14] Lee J, Quraishi SA, Bhatnagar S, Zafonte RD, Masiakos PT. The economic cost of firearm-related injuries in the United States from 2006 to 2010. Surgery. 2014;155(5):894-898. doi:10.1016/j.surg.2014.02.011 24684950

[15] von Elm E, Altman DG, Egger M, Pocock SJ, Gøtzsche PC, Vandenbroucke JP; STROBE Initiative. The Strengthening the Reporting of Observational Studies in Epidemiology (STROBE) statement: guidelines for reporting observational studies. J Clin Epidemiol. 2008;61(4):344-349. doi:10.1016/j.jclinepi.2007.11.008 18313558

[16] Husereau D, Drummond M, Augustovski F, . Consolidated Health Economic Evaluation Reporting Standards 2022 (CHEERS 2022) statement: updated reporting guidance for health economic evaluations. MDM Policy Pract. 2022;7(1):23814683211061097. doi:10.1177/23814683211061097 35036563 PMC8755935

[17] Healthcare Cost and Utilization Project. American Hospital Association linkage files. Accessed December 30, 2024. https://hcup-us.ahrq.gov/reports/methods/methods.jsp

[18] Kralovec PD, Mullner R. The American Hospital Association’s Annual Survey of Hospitals: continuity and change. Health Serv Res. 1981;16(3):351-355. 7298343 PMC1072253

[19] CMS Office of Minority Health. CMS framework for health equity 2022–2032. Accessed February 14, 2025. https://www.cms.gov/files/document/cms-framework-health-equity.pdf

[20] Federal Reserve Bank of Minneapolis. Inflation calculator. Accessed December 30, 2024, https://www.minneapolisfed.org/about-us/monetary-policy/inflation-calculator

[21] Hedegaard H, Johnson RL, Garnett MF, Thomas KE. The *2020 International Classification of Diseases, 10th Revision, Clinical Modification* injury diagnosis framework for categorizing injuries by body region and nature of injury. Natl Health Stat Report. 2020;(150):1-27. 33395385

[22] Clark DE, Black AW, Skavdahl DH, Hallagan LD. Open-access programs for injury categorization using *ICD-9* or *ICD-10*. Inj Epidemiol. 2018;5(1):11. doi:10.1186/s40621-018-0149-8 29629480 PMC5890002

[23] Menendez ME, Ring D. A comparison of the Charlson and Elixhauser comorbidity measures to predict inpatient mortality after proximal humerus fracture. J Orthop Trauma. 2015;29(11):488-493. doi:10.1097/BOT.0000000000000380 26165266

[24] Shaka H, Edigin E. A revised comorbidity model for administrative databases using clinical classifications software refined variables. Cureus. 2021;13(12):e20407. doi:10.7759/cureus.20407 35047250 PMC8756739

[25] Thomas AC, Royan R, Nathens AB, . Patient and hospital characteristics associated with admission among patients with minor isolated extremity firearm injuries: a propensity-matched analysis. Ann Surg Open. 2024;5(2):e430. doi:10.1097/AS9.0000000000000430 38911659 PMC11191909

[26] Smart R, Peterson S, Schell TL, Kerber R, Morral AR. Inpatient Hospitalizations for Firearm Injury: Estimating State-Level Rates From 2000 to 2016. RAND; 2021. 10.7249/rhq-09-04-10 PMC9519112 36238005

[27] Barber C, Hemenway D. Too many or too few unintentional firearm deaths in official U.S. mortality data? Accid Anal Prev. 2011;43(3):724-731. doi:10.1016/j.aap.2010.10.018 21376860

[28] Cook PJ, Rivera-Aguirre AE, Cerdá M, Wintemute G. Constant lethality of gunshot injuries from firearm assault: United States, 2003–2012. Am J Public Health. 2017;107(8):1324-1328. doi:10.2105/AJPH.2017.303837 28640677 PMC5508146

[29] Hink AB, Bonne S, Levy M, . Firearm injury research and epidemiology: a review of the data, their limitations, and how trauma centers can improve firearm injury research. J Trauma Acute Care Surg. 2019;87(3):678-689. doi:10.1097/TA.0000000000002330 31033891

[30] Spitzer SA, Forrester JD, Tennakoon L, Spain DA, Weiser TG. A decade of hospital costs for firearm injuries in the United States by region, 2005-2015: government healthcare costs and firearm policies. Trauma Surg Acute Care Open. 2022;7(1):e000854. doi:10.1136/tsaco-2021-000854 35497324 PMC8995943

[31] Okeke G, Sana M, Faridmoayer E, Kougias P, Sharath SE. Financial burden and outcomes of firearm injuries in U.S. hospitals, 2003-2020. Am J Prev Med. 2025;68(1):75-82. doi:10.1016/j.amepre.2024.08.021 39218408

[32] Spitzer SA, Staudenmayer KL, Tennakoon L, Spain DA, Weiser TG. Costs and financial burden of initial hospitalizations for firearm injuries in the United States, 2006–2014. Am J Public Health. 2017;107(5):770-774. doi:10.2105/AJPH.2017.303684 28323465 PMC5388949

[33] Healthcare Cost and Utilization Project. HCUP fact sheet. Accessed December 30, 2024. https://hcup-us.ahrq.gov/news/exhibit_booth/hcup_fact_sheet.jsp

[34] Healthcare Cost and Utilization Project. ZIPINC_QRTL: median household income for patient's ZIP Code (based on current year). Accessed August 28, 2025. https://hcup-us.ahrq.gov/db/vars/siddistnote.jsp?var=zipinc_qrtl

[35] Abrams DS. COVID and crime: an early empirical look. J Public Econ. 2021;194:104344. doi:10.1016/j.jpubeco.2020.104344 33518828 PMC7826063

[36] Kim DY, Phillips SW. When COVID-19 and guns meet: a rise in shootings. J Crim Justice. 2021;73:101783. doi:10.1016/j.jcrimjus.2021.101783 33518825 PMC7825997

[37] Andrews AL, Killings X, Oddo ER, Gastineau KAB, Hink AB. Pediatric firearm injury mortality epidemiology. Pediatrics. 2022;149(3):e2021052739. doi:10.1542/peds.2021-052739 35224633

[38] Iantorno SE, Swendiman RA, Bucher BT, Russell KW. Surge in pediatric firearm injuries presenting to US children’s hospitals during the COVID-19 pandemic. JAMA Pediatr. 2023;177(2):204-206. doi:10.1001/jamapediatrics.2022.4881 36534391 PMC9856622

[39] Miller M, Azrael D, Yenduri R, . Assessment of the accuracy of firearm injury intent coding at 3 US hospitals. JAMA Netw Open. 2022;5(12):e2246429. doi:10.1001/jamanetworkopen.2022.46429 36512356 PMC9856424

[40] Shayan M, Lew D, Mancini M, Foraker RE, Doering M, Mueller KL. A systematic review of recurrent firearm injury rates in the United States. Prev Med. 2023;168:107443. doi:10.1016/j.ypmed.2023.107443 36740145

[41] Price MD, McDermott KM, Gorijavolu R, . Pediatric firearm reinjury: a retrospective statewide risk factor analysis. J Surg Res. 2024;303:568-578. doi:10.1016/j.jss.2024.09.066 39427472 PMC11665736

[42] Chong VE, Smith R, Garcia A, . Hospital-centered violence intervention programs: a cost-effectiveness analysis. Am J Surg. 2015;209(4):597-603. doi:10.1016/j.amjsurg.2014.11.003 25728889

[43] O’Toole MJ, Schnippel K, Larson B. Hospital-based violence intervention programs: an analysis of costs and key components. J Trauma Acute Care Surg. 2025;98(4):655-661. 40122847 10.1097/TA.0000000000004498

[44] Balasubramanian SS, Jones EC. Hospital closures and the current healthcare climate: the future of rural hospitals in the USA. Rural Remote Health. 2016;16(3):3935. doi:10.22605/RRH3935 27466156

[45] Kaufman BG, Thomas SR, Randolph RK, . The rising rate of rural hospital closures. J Rural Health. 2016;32(1):35-43. doi:10.1111/jrh.12128 26171848

[46] Buchmueller TC, Jacobson M, Wold C. How far to the hospital? the effect of hospital closures on access to care. J Health Econ. 2006;25(4):740-761. doi:10.1016/j.jhealeco.2005.10.006 16356570

[47] McCarthy S, Moore D, Smedley WA, . Impact of rural hospital closures on health-care access. J Surg Res. 2021;258:170-178. doi:10.1016/j.jss.2020.08.055 33011448

[48] Bell N, Hung P, Merrell MA, Crouch E, Eberth JM. Changes in access to community health services among rural areas affected and unaffected by hospital closures between 2006 and 2018: a comparative interrupted time series study. J Rural Health. 2023;39(1):291-301. doi:10.1111/jrh.12691 35843725

[49] Lindrooth RC, Perraillon MC, Hardy RY, Tung GJ. Understanding the relationship between Medicaid expansions and hospital closures. Health Aff (Millwood). 2018;37(1):111-120. doi:10.1377/hlthaff.2017.0976 29309219

[50] Peterson C, Xu L, Florence C, Grosse SD, Annest JL. Professional fee ratios for US hospital discharge data. Med Care. 2015;53(10):840-849. doi:10.1097/MLR.0000000000000410 26340662 PMC4681390

[51] Madiraju SK, Catino J, Kokaram C, Genuit T, Bukur M. In by helicopter out by cab: the financial cost of aeromedical overtriage of trauma patients. J Surg Res. 2017;218:261-270. doi:10.1016/j.jss.2017.05.102 28985859

[52] Rattan R, Parreco J, Namias N, Pust GD, Yeh DD, Zakrison TL. Hidden costs of hospitalization after firearm injury: national analysis of different hospital readmission. Ann Surg. 2018;267(5):810-815. doi:10.1097/SLA.0000000000002529 28922206

[53] Song Z, Zubizarreta JR, Giuriato M, Paulos E, Koh KA. Changes in health care spending, use, and clinical outcomes after nonfatal firearm injuries among survivors and family members : a cohort study. Ann Intern Med. 2022;175(6):795-803. doi:10.7326/M21-2812 35377713

[54] Miller T, Downing J, Wheeler L, Fischer K. The medical costs of firearm injuries in the United States: a systematic review. J Emerg Med. 2024;66(2):109-132. doi:10.1016/j.jemermed.2023.08.013 38262782

