# Errors in Text, Tables, and Figure

**DOI:** 10.1001/jamahealthforum.2025.5429

**Published:** 2025-11-07

In the Original Investigation titled “Health Care Costs of Firearm Injury Hospital Visits in the US,”^1^ published online September 26, 2025, there were errors in the text, Tables 1 and 2, and Figure 3. In the Discussion, Tables 1 and 2, and Figure 3, information on hospital community violence prevention programs was inadvertently included and removed after publication at the authors’ request. Additionally, in the Total Cost subsection in the Results, the text was updated to “Hospital costs of care were $4.0 billion for patients with Medicaid as payer, ….” This article was corrected online.
