# Supplementary material for: Health Care Costs of Firearm Injury Hospital Visits in the US
Source: JAMA Health Forum. 2025 Sep 26;6(9):e253299. doi: 10.1001/jamahealthforum.2025.3299 (PMC12475945; doi:10.1001/jamahealthforum.2025.3299)
Supplement: Supplement 1. — eMethods. Monte Carlo Simulation R Code eTable 1. List of International Statistical Classification of Diseases and Related Health Problems, Tenth Revision Codes to Identify Firearm Injury Patients eTable 2. Monte Carlo Estimates for Median Hospital Cost per Emergency Department and Inpatient Visits for New Firearm Injuries in the US from 2016 to 2021 eFigure. National Monte Carlo Estimates of Mean Cost for Firearm Injury Emergency Department Visits and Inpatient Hospitalizations by Body Region [file jamahealthforum-e253299-s001.pdf]

Supplemental Online Content

Royan R, Lundberg A, Shan Y, Thomas AC, Stey AM. Health care costs of firearm injury hospital visits in the US. *JAMA Health Forum*. 2025;6(9):e253299. doi:10.1001/jamahealthforum.2025.3299

eMethods. Monte Carlo Simulation R Code

eTable 1. List of *International Statistical Classification of Diseases and Related Health Problems, Tenth Revision* Codes to Identify Firearm Injury Patients

eTable 2. Monte Carlo Estimates for Median Hospital Cost per Emergency Department and Inpatient Visits for New Firearm Injuries in the US from 2016 to 2021

eFigure. National Monte Carlo Estimates of Mean Cost for Firearm Injury Emergency Department Visits and Inpatient Hospitalizations by Body Region

This supplementary material has been provided by the authors to give readers additional information about their work.

© 2025 Royan R et al. *JAMA Health Forum*.

eMethods. Monte Carlo Simulation R Code

```
#setwd
rm(list=ls())
setwd("C:/Users/all7053/OneDrive - Northwestern University/firearm/data")
#note: sedd ccr 2017 does not contain NY, sid ccr 2018 does not contain NY

#packages
library(haven)
library(tidyr)
library(dplyr)
library(icdpicr)
library(data.table)

#firearm injuries
df_full=read.csv("firearm_iss.csv")

#population
pop=read.csv("population.csv")
pop = pop %>%
pivot_longer(cols=pop_2016:pop_2021,names_to="year",names_prefix="pop_",values_to="popula
tion")

#rand estimates
rand=read.csv("updated_rand/nonfatal_predictions_cc_forAlex.csv")
rand=rand[,names(rand) %in% c("state","year","pred_nf10K_mean")]

#merge population from Census
rand=merge(rand,pop)

#inpatient visits
rand$inpat=round(rand$population*rand$pred_nf10K_mean/10000)

#replace blanks
df_full[df_full==""]=NA

#recode state abbreviations to match rand
df_full$state=recode(df_full$HOSPST,AR="Arkansas",FL="Florida",MA="Massachusetts",MD="Mar
yland",NY="New York",WI="Wisconsin")

#merge in hospital data
aha_2016=read_sas("R:/Surgery/Trauma-CriticalCare/Stey_Lab/AHA/2016 Annual Survey
(2018)/aha2016.sas7bdat")
```

© 2025 Royan R et al. *JAMA Health Forum*.

```
aha_2018=read_sas("R:/Surgery/Trauma-CriticalCare/Stey_Lab/AHA/2018 Annual Survey
(2020)/FLAT/AS2018PUB.sas7bdat")

hosp_vars=c("ID","HOSPBD","CBSATYPE","MCDDC","ADMTOT","TRAUMHOS","TRAUML90","RURL
HOS","VCMMHOS","ADMTOT","VEM","MAPP5")
aha_2016 = aha_2016 %>% select(hosp_vars)
aha_2018 = aha_2018 %>% select(hosp_vars)

names(df_full)[names(df_full)=="AHAID"]="ID"
df_full_2016=merge(df_full[df_full$YEAR %in% c(2016,2017),],aha_2016,by="ID",all.x=T)
df_full_2018=merge(df_full[df_full$YEAR %in%
c(2018,2019,2020,2021),],aha_2016,by="ID",all.x=T)
df_full=rbind(df_full_2016,df_full_2018)
df_full[df_full==""]=NA

#get hospital variables for simulation
df_full$medicaid_rate=df_full$MCDDC/df_full$ADMTOT

#get costs from cost-to-charge ratio
df_full$APECC=as.numeric(df_full$APECC)
df_full$APICC=as.numeric(df_full$APICC)
df_full$ccr=ifelse(is.na(df_full$APECC),df_full$APICC,df_full$APECC)
df_full$cost=df_full$ccr*df_full$TOTCHG

#get repeat visit indicator
df_full = df_full %>% group_by(state) %>%
mutate(repeat_visit=as.integer(duplicated(VisitLink,incomparables=NA)))

#proportion ED-only in sample
ed_ratio=sum(df_full$sedd==1)/sum(df_full$sedd==0)

#reduce data frame to save computation demands
hosp_vars=c("HOSPBD","CBSATYPE","medicaid_rate","TRAUMHOS","TRAUML90","RURLHOS","VC
MMHOS","ADMTOT","VEM","MAPP5")
vars=c("sedd","state","YEAR","VisitLink","AGE","FEMALE","HISPANIC","RACE","PAY1","ZIPINC_QR
TL","Homeless","elix_visit","intent","DISPUNIFORM",

"mxaisbr_General_visit","mxaisbr_HeadNeck_visit","mxaisbr_Face_visit","mxaisbr_Extremities_visi
t","mxaisbr_Chest_visit","mxaisbr_Abdomen_visit","maxais_visit","riss_visit",
    "cost","repeat_visit")
vars=c(vars,hosp_vars)

df=df_full[,names(df_full) %in% vars]
#cost missing for 16% of sample
```

© 2025 Royan R et al. *JAMA Health Forum*.

```
df=df[!is.na(df$cost),]
#clean memory
df=as.data.frame(df)
gc()

#separate inpatient and ED-only
sedd=df[df$sedd==1,]
sid=df[df$sedd==0,]

### simulation ###
n_sim=2400
batch=50
set.seed(100)
list_sim=list()

setwd("C:/Users/all7053/OneDrive - Northwestern University/firearm/sim")

#kick out our states from RAND
rand=rand[!rand$state %in% unique(df$state),]

#start loop
for(s in 1:batch){
for(i in 1:(n_sim/batch)){

sim_df=data.frame(matrix(ncol=ncol(df),nrow=0))

#simulate states with rand estimates
states=unique(rand$state)
years=sort(unique(df_full$YEAR))
for(j in 1:length(states)){
 for(k in 1:length(years)){
   n_in=rand$inpat[rand$state==states[j] & rand$year==years[k]]
   n_ed=round(ed_ratio*n_in)
   in_sim=sample_n(sid[sid$YEAR==years[k],],n_in,replace=T)
   ed_sim=sample_n(sedd[sedd$YEAR==years[k],],n_ed,replace=T)
   sim_df=rbind(sim_df,rbind(in_sim,ed_sim))
 }
}

#simulate missing values in our own states
states=c("Florida","Arkansas","Maryland","Massachusetts","New York","Wisconsin")
years=sort(unique(df_full$YEAR))
for(j in 1:length(states)){
 for(k in 1:length(years)){
```

© 2025 Royan R et al. *JAMA Health Forum*.

```
   n_in=sum(is.na(df_full$cost[df_full$state==states[j] & df_full$sedd==0 &
df_full$YEAR==years[k]]))
   n_ed=sum(is.na(df_full$cost[df_full$state==states[j] & df_full$sedd==1 &
df_full$YEAR==years[k]]))
   in_sim=sample_n(sid[sid$state==states[j] & sid$YEAR==years[k],],n_in,replace=T)
   ed_sim=sample_n(sedd[sedd$state==states[j] & sedd$YEAR==years[k],],n_ed,replace=T)
   sim_df=rbind(sim_df,rbind(in_sim,ed_sim))
  }
}

list_sim[[i]]=sim_df
list_sim[[i]]$sim_no=rep((s-1)*n_sim/batch+i,nrow(sim_df))
}

print(s)
sim=bind_rows(list_sim)
fwrite(sim,paste0("sim_",s,".csv"))
rm(sim)
gc()

}
```

© 2025 Royan R et al. *JAMA Health Forum*.

eTable 1. List of *International Statistical Classification of Diseases and Related Health Problems, Tenth Revision* Codes to Identify Firearm Injury Patients

W320XXA, W321XXA, W3300XA, W3301XA, W3302XA, W3303XA, W3309XA, W3310XA, W3311XA, W3312XA, W3313XA, W3319XA, W3400XA, W3409XA, W3410XA, W3419XA, X72XXXA, X730XXA,
X731XXA, X732XXA, X738XXA, X739XXA, X748XXA, X749XXA, X93XXXA, X940XXA, X941XXA, X942XXA, X948XXA, X949XXA, X958XXA, X959XXA, Y22XXXA, Y230XXA, Y231XXA, Y232XXA, Y233XXA, Y238XXA,
Y239XXA, Y248XXA, Y249XXA, Y35001A, Y35002A,Y35003A, Y35009A, Y35011A, Y35012A, Y35013A, Y35019A, Y35021A,Y35022A, Y35023A,Y35029A, Y35031A, Y35032A, Y35033A, Y35039A, Y35091A, Y35092A, Y35093A, Y35099A

© 2025 Royan R et al. *JAMA Health Forum*.

eTable 2. Monte Carlo Estimates for Median Hospital Cost per Emergency Department and Inpatient Visits for New Firearm Injuries in the US from 2016 to 2021

	Emergency Department N=298,721		Inpatient N=185,846	
	**Median Cost[a]**	**IQR**	**Median Cost[a]**	**IQR**
**Overall**	1,028	4.2	21,385	85.6
**Age**				
0-17	905	11.1	21,777	302.8
18-24	1,058	5.8	21,094	208.4
25-44	1,072	6.4	22,532	132.5
45-64	941	13.7	20,205	233.6
65+	848	17.0	16,503	439.6
**Sex**				
Male	1,036	4.9	21,601	86.5
Female	954	9.5	20,002	118.8
**Race/ethnicity[b]**				
White	763	5.2	18,676	164.2
Black	1,142	6.2	22,588	104.2
Hispanic	1,026	6.1	21,014	149.0
Asian/Pac. Isl.	922	21.2	21,368	1,290.3
Native American	1,220	149.4	16,873	264.3
Other	1,206	21.8	24,086	728.8
**Insurance**				
Medicaid	1,136	4.8	24,831	136.4
Medicare	771	6.0	17,710	323.5
Private	874	9.5	20,571	150.2
Self-Pay	1,067	8.0	17,518	174.3
No Charge[c]	818	27.7	18,565	201.2
Other[d]	1,052	34.5	21,657	496.4
**ZIP Income Qrt[e]**				
1	1,031	4.3	21,730	105.5
2	962	11.3	20,343	108.6
3	1,104	9.1	21,980	177.4
4	995	9.6	22,085	360.2
**Discharge Disposition**				
Routine	946	4.8	18,638	51.2
Transfer to Acute Care Hospital	972	7.6	26,327	723.9
Transfer to Other Facility[f]	1,051	15.6	51,137	783.1
Home Health Care	2,958	35.9	34,945	656.6

© 2025 Royan R et al. *JAMA Health Forum*.

Against Medical Advice	1,015	15.9	12,689	386.4
Died	1,632	14.0	20,458	205.4
**Intent**				
Assault	1,466	5.5	24,055	128.8
Self-Inflicted	1,425	48.3	18,804	325.8
Unintentional	839	3.7	19,403	93.5
Undetermined	1,264	26.7	17,424	272.7
Legal Intervention	641	13.7	28,578	502.6
**Body Region**				
Head and neck	1,167	16.1	14,681	186.5
Face	763	21.1	21,014	336.3
Chest	1,423	22.0	18,331	298.5
Abdomen	1,627	16.4	24,155	174.6
Extremities	789	3.8	15,184	96.8
Multiple Regions	1,892	16.9	30,285	149.4
Other[g]	627	15.3	5,829	192.9
**Injury Severity Score**				
0-8	883	3.3	12,864	51.7
9-25	1,374	10.1	23,935	115.0
26-75	2,022	37.6	42,398	309.4
**Elixhauser Comorbidity Score**				
0	955	3.8	16,130	67.9
1	1,295	13.7	20,925	77.0
2	1,479	18.2	27,603	234.9
3+	1,807	41.3	39,934	470.4
**Reinjury[h]**				
0	1,047	4.1	21,570	94.2
1+	878	10.4	20,097	186.9
**Hospital Beds**				
1-99	663	7.9	11,355	283.3
100-499	814	2.5	17,257	103.9
500+	1,615	7.3	24,433	101.1
**Hospital Medicaid %[i]**				
Quartile 1	752	4.4	17,301	170.5
Quartile 2	854	6.5	19,766	139.1
Quartile 3	1,416	13.6	23,515	164.4
Quartile 4	1,266	6.9	24,578	256.5
**Core-Based Statistical Area**				
Metro	1,069	3.9	21,501	99.8

© 2025 Royan R et al. *JAMA Health Forum*.

Micro	632	5.9	11,871	156.9
Rural	713	9.3	10,738	1,568.7
**Level of Trauma Center**				
Regional Resource	1,823	9.5	25,441	124.0
Community	1,188	8.8	19,786	152.9
Rural	747	4.6	13,629	266.5
Greater	697	11.7	10,131	913.3
**Rural Hospital**				
No	1,093	3.7	22,212	138.9
Yes	927	13.3	19,929	193.9
**Total Admissions**[i]				
Quartile 1	759	8.3	17,294	213.9
Quartile 2	901	16.0	17,956	136.2
Quartile 3	1,668	42.5	24,052	266.4
Quartile 4	1,429	19.6	23,723	174.4
**Total ED Visits**[i]				
Quartile 1	710	5.3	16,029	120.1
Quartile 2	1,091	5.2	22,588	217.2
Quartile 3	1,149	7.6	23,143	225.6
Quartile 4	1,442	24.7	22,951	236.6
**Medical School Affiliation**				
No	682	2.4	15,591	109.6
Yes	1,323	6.0	23,815	92.5

a. Dollars are inflation-adjusted to the year 2022.

b. In the race/ethnicity variable code, ethnicity takes precedence over race. Black and White, for example, therefore refer to Non-Hispanic Black and Non-Hispanic White.

c. Arkansas and Wisconsin classify no charge patients as self-pay.

d. Other Insurance includes Worker's Compensation, CHAMPUS, CHAMPVA, Title V, and other government programs.

e. Zip code income quartile is a quartile classification of the estimated median household income of residents in the patient's ZIP Code.

f. Other type of facility includes discharge to hospice, rehabilitation, Long-Term Care Hospital, psychiatric hospital, skilled nursing facility, or intermediate care facility.

g. Other includes those unclassifiable by body site using the Barell Matrix.

h. Re-injury is defined as the number of times a patient presented during the year for a new firearm injury.

i. See the Methods for the median and interquartile range of the quartile variables. Hospital Medicaid % is calculated as the ratio of Medicaid discharges to total discharges in ascending order.

© 2025 Royan R et al. *JAMA Health Forum*.

eFigure. National Monte Carlo Estimates of Mean Cost for Firearm Injury Emergency Department Visits and Inpatient Hospitalizations by Body Region

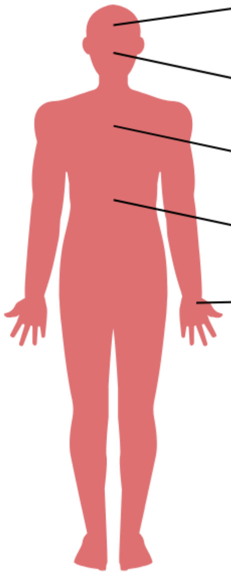

	Treated in the Emergency Department and Discharged		Inpatient Hospitalization	
	Mean Cost per Injury	Percent of Total Costs	Mean Cost per Injury	Percent of Total Costs
Head and Neck	$1,659	7.54%	$32,062	3.7%
Face	$1,511	1.50%	$35,935	1.3%
Chest	$2,065	7.23%	$30,871	3.7%
Abdominal	$2,345	9.00%	$38,911	10.5%
Extremities	$1,375	44.14%	$20,944	15.4%
Other	$1,,049	0.45%	$11,746	0.9%
Multiple Regions	$2,668	30.15%	$53,856	64.4%
Average Cost Any Body Area	$1,743		$38,879	
Total Cost for ED Care in Discharged Patients	$520,663,228			
Total Cost for Inpatient Care in Admitted Patients			$7,226,687,268	

© 2025 Royan R et al. *JAMA Health Forum*.
