# Supplementary material for: Health Care Costs of Firearm Injury Hospital Visits in the US
Source: JAMA Health Forum. 2025 Sep 26;6(9):e253299. doi: 10.1001/jamahealthforum.2025.3299 (PMC12475945; doi:10.1001/jamahealthforum.2025.3299)
Supplement: Supplement 2. — Data Sharing Statement [file jamahealthforum-e253299-s002.pdf]

Data Sharing Statement

Royan. Health Care Costs of Firearm Injury Hospital Visits in the US. *JAMA Health Forum*. Published September 26, 2025. doi:10.1001/jamahealthforum.2025.3299

Data
Data available: No

Additional Information
Explanation for why data not available: We cannot share the HCUP data because of data use agreements, but we have included replication code, and other researchers can purchase the data through AHRQ.
